# Supplementary material for: Msn2/4 regulate expression of glycolytic enzymes and control transition from quiescence to growth
Source: eLife. 2017 Sep 26;6:e29938. doi: 10.7554/eLife.29938 (PMC5634782; doi:10.7554/eLife.29938)
Supplement: Transparent reporting form [file elife-29938-transrepform.docx]

***eLife’s* transparent reporting form**

We encourage authors to provide detailed information *within their submission* to facilitate the interpretation and replication of experiments. If you have any questions, please contact us: [editorial@elifesciences.org](mailto:editorial@elifesciences.org).

**Sample-size estimation**

- You should state whether an appropriate sample size was computed when the study was being designed
- You should state the statistical method of sample size computation and any required assumptions
- If no explicit power analysis was used, you should describe how you decided what sample (replicate) size (number) to use

Please outline where this information can be found within the submission (e.g., page numbers or figure legends), or explain why this information doesn’t apply to your submission:

We didn’t perform explicit power analysis. Major findings stem from time-course analysis of genomic profiling and we described the rationale of sampling in the second paragraph of section “Msn2/4 regulates expression of the glycolytic pathway and accumulation of acetyl-CoA” on page 7 line 6-8 and in the ChIP-seq section of materials and methods on page 15 line 1-3.

**Replicates**

- You should report how often each experiment was performed
- You should include a definition of biological versus technical replication
- The data obtained should be provided and sufficient information should be provided to indicate the number of independent biological and/or technical replicates
- If you encountered any outliers, you should describe how these were handled
- Criteria for exclusion/inclusion of data should be clearly stated
- High-throughput sequence data should be uploaded before submission, with a private link for reviewers provided (these are available from both GEO and ArrayExpress)

Please outline where this information can be found within the submission (e.g., page numbers or figure legends), or explain why this information doesn’t apply to your submission:

Replication information can be found in the materials and methods section under subtitles of “Metabolic cycles”, “Acetyl-CoA measurement”, “Yeast survival measurements” and “Cell size measurement” on page 13 L11, page 16 L9-19, page 16 L24, page 17 L3. High-throughput sequence data information can be found in the “Data accession” section on page 19 L23-25.

**Statistical reporting**

- Statistical analysis methods should be described and justified
- Raw data should be presented in figures whenever informative to do so (typically when N per group is less than 10)
- For each experiment, you should identify the statistical tests used, exact values of N, definitions of center, methods of multiple test correction, and dispersion and precision measures (e.g., mean, median, SD, SEM, confidence intervals; and, for the major substantive results, a measure of effect size (e.g., Pearson's r, Cohen's d)
- Report exact p-values wherever possible alongside the summary statistics and 95% confidence intervals. These should be reported for all key questions and not only when the p-value is less than 0.05.

Statistical analysis in the DynaMO method and its application can be found in the Materials and methods section and the related manuscript “Dynamic motif occupancy (DynaMO) analysis identifies transcription factors and their bidning sites driving dynamic biological processes”. Dispersion measures for Figure 3-Figure Supplement 2 and 3 can be found in the figure legends on page 24-25

Please outline where this information can be found within the submission (e.g., page numbers or figure legends), or explain why this information doesn’t apply to your submission:

(For large datasets, or papers with a very large number of statistical tests, you may upload a single table file with tests, Ns, etc., with reference to page numbers in the manuscript.)

**Additional data files (“source data”)**

- We encourage you to upload relevant additional data files, such as numerical data that are represented as a graph in a figure, or as a summary table
- Where provided, these should be in the most useful format, and they can be uploaded as “Source data” files linked to a main figure or table
- Include model definition files including the full list of parameters used
- Include code used for data analysis (e.g., R, MatLab)
- Avoid stating that data files are “available upon request”

Please indicate the figures or tables for which source data files have been provided:

Source data files for Figure 1, 2 and 4 are provided. Source code is included and can be found on https://github.com/spo111/DynaMO.
